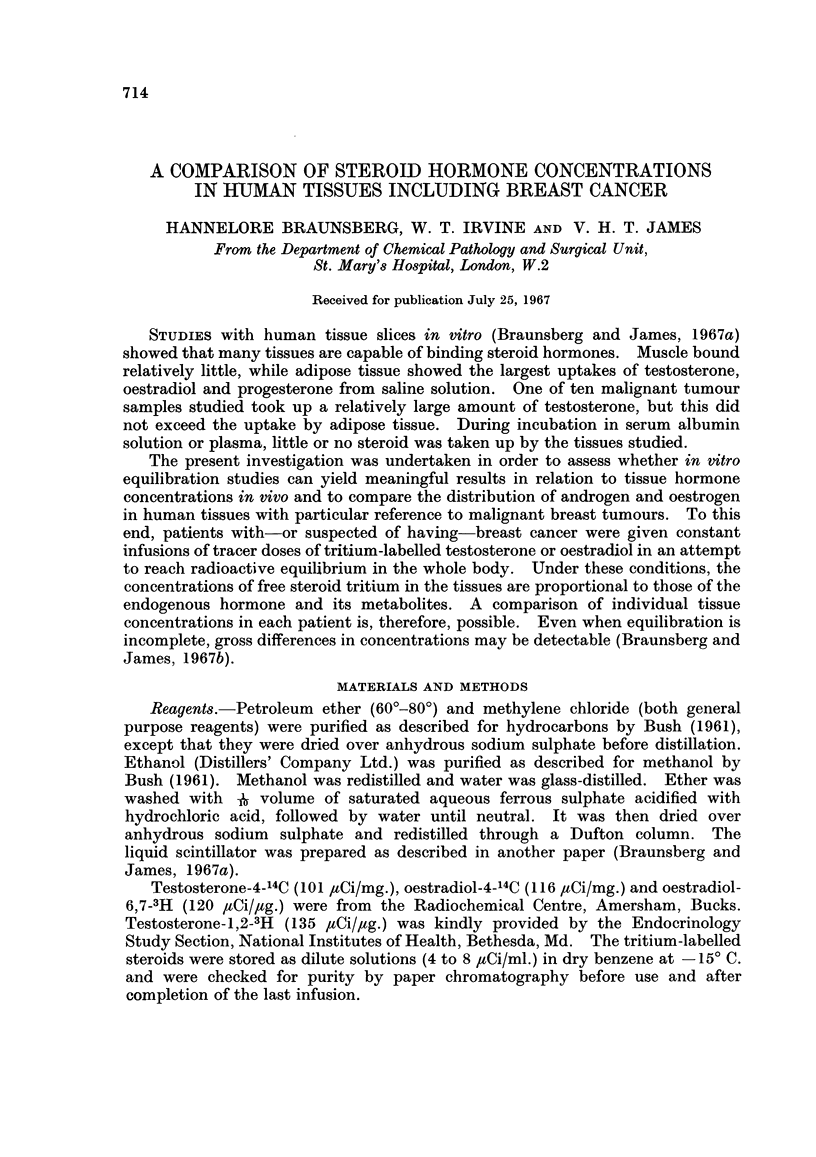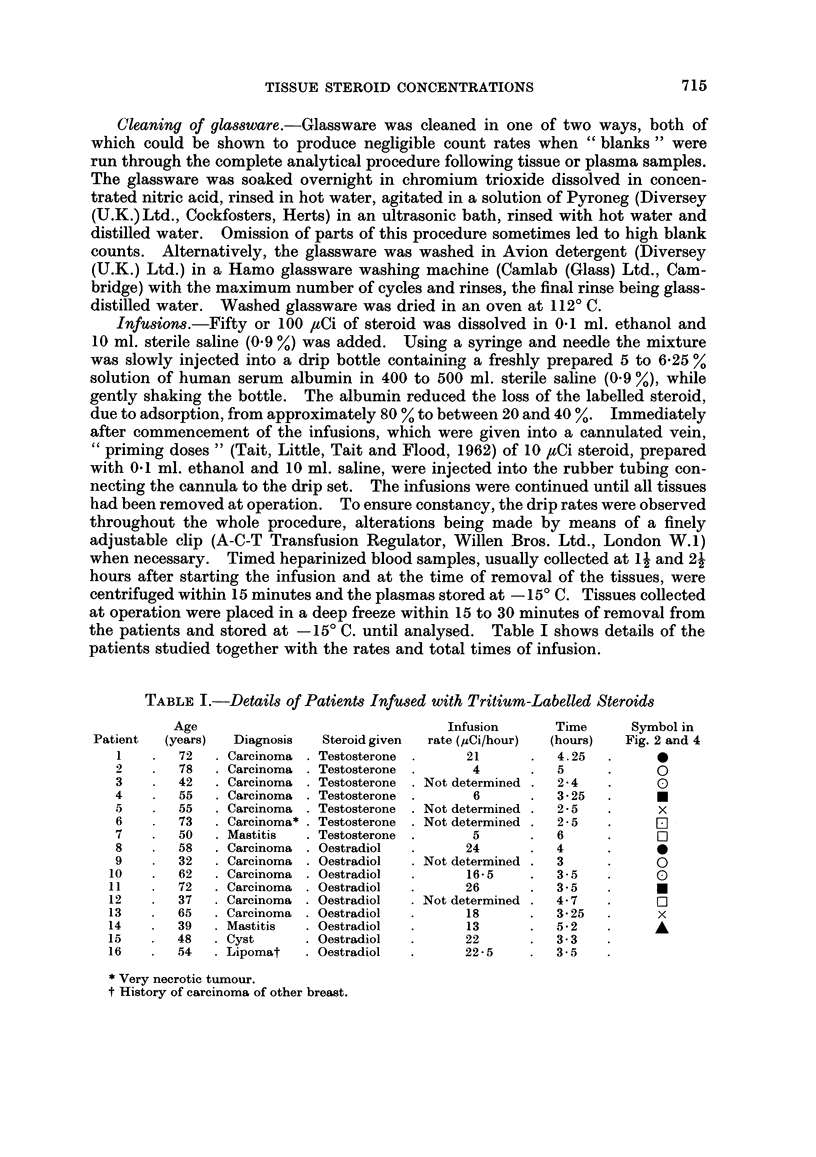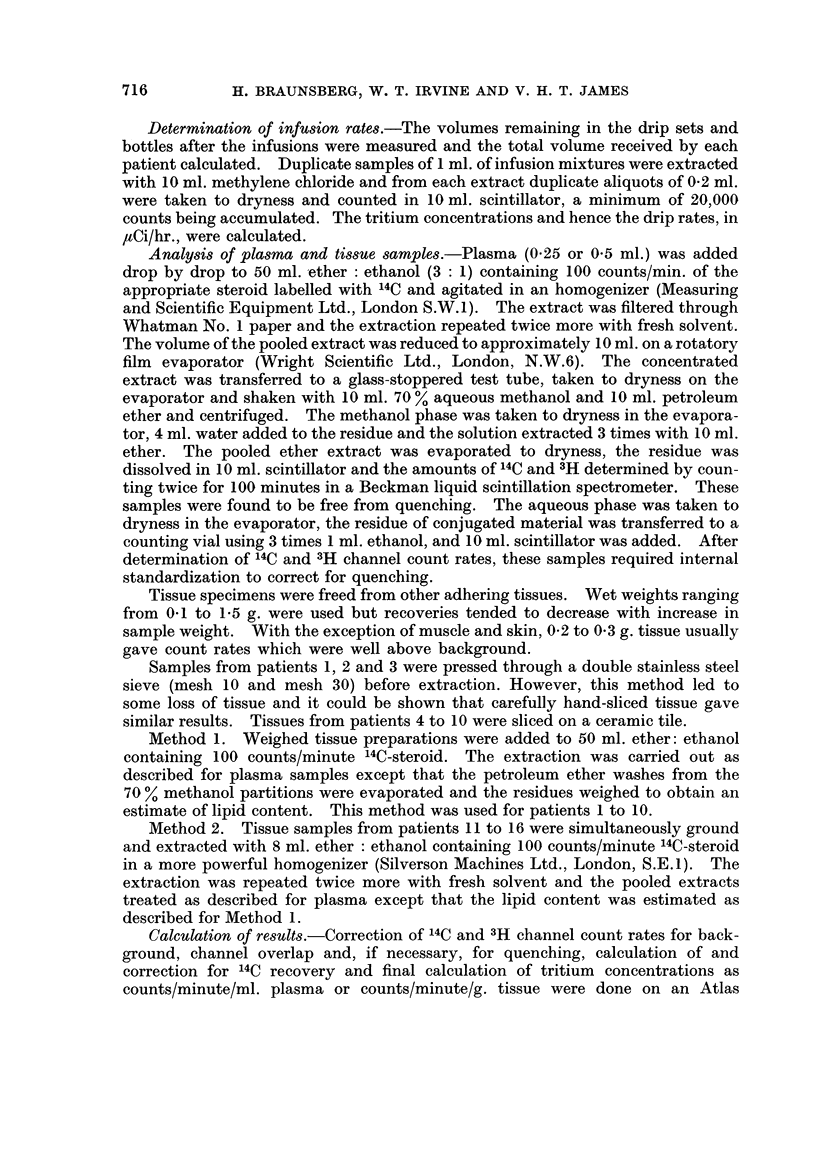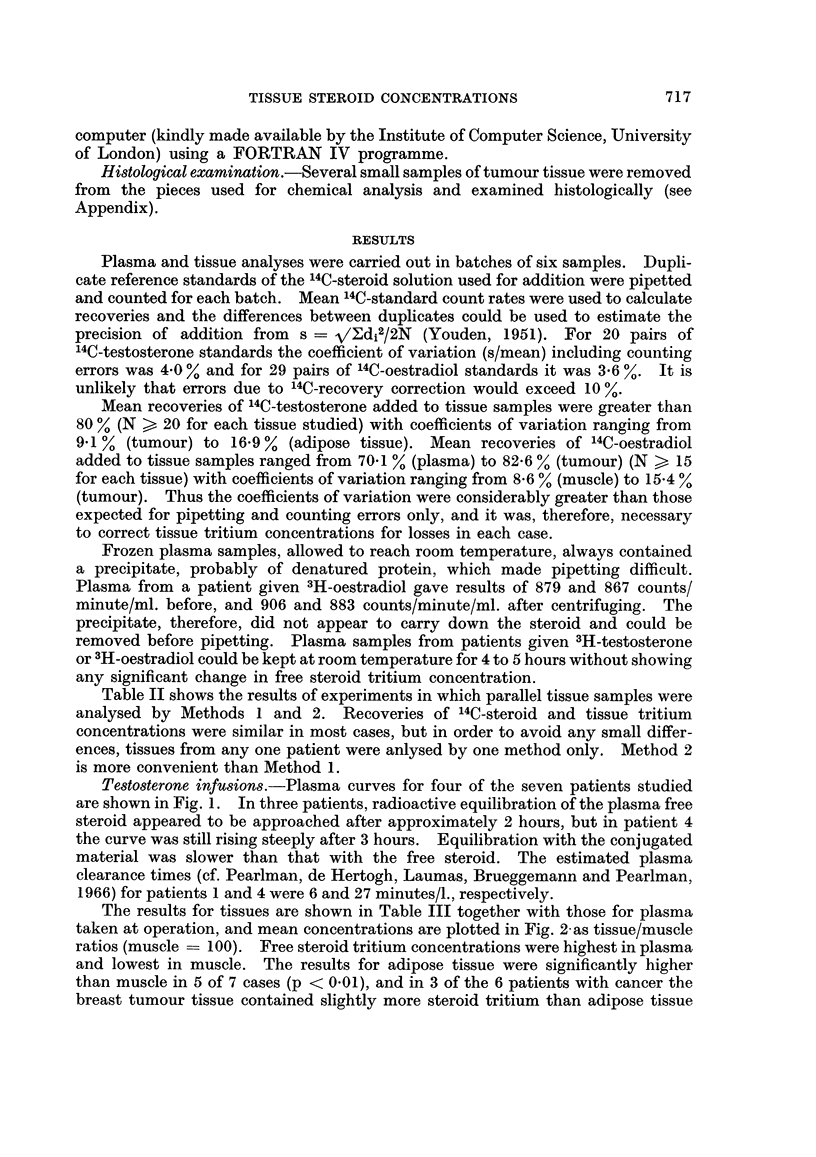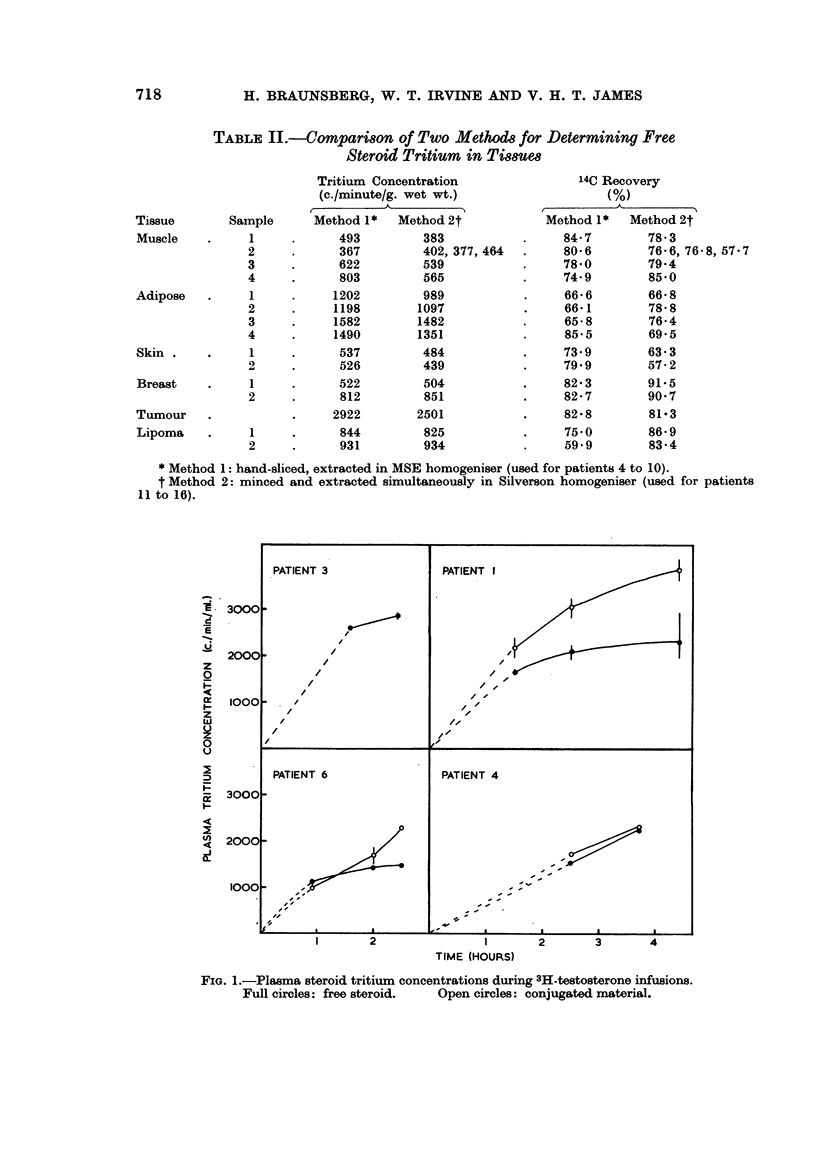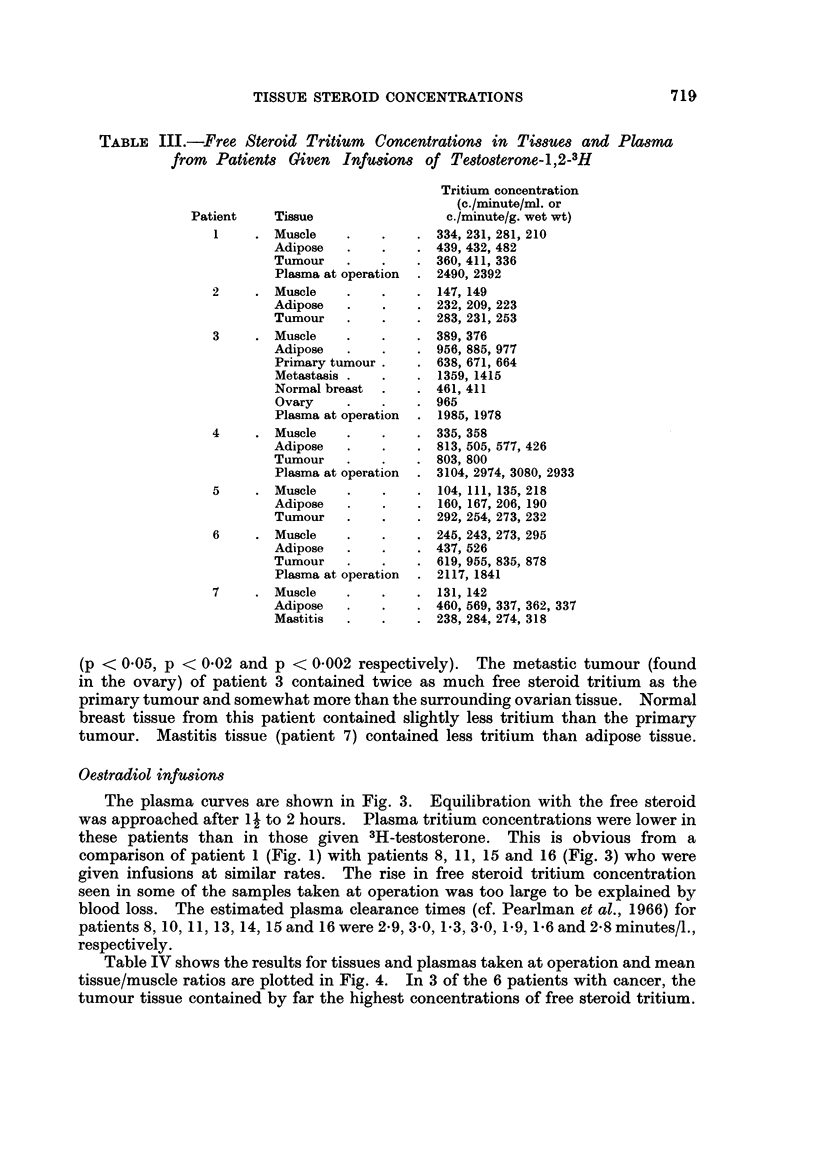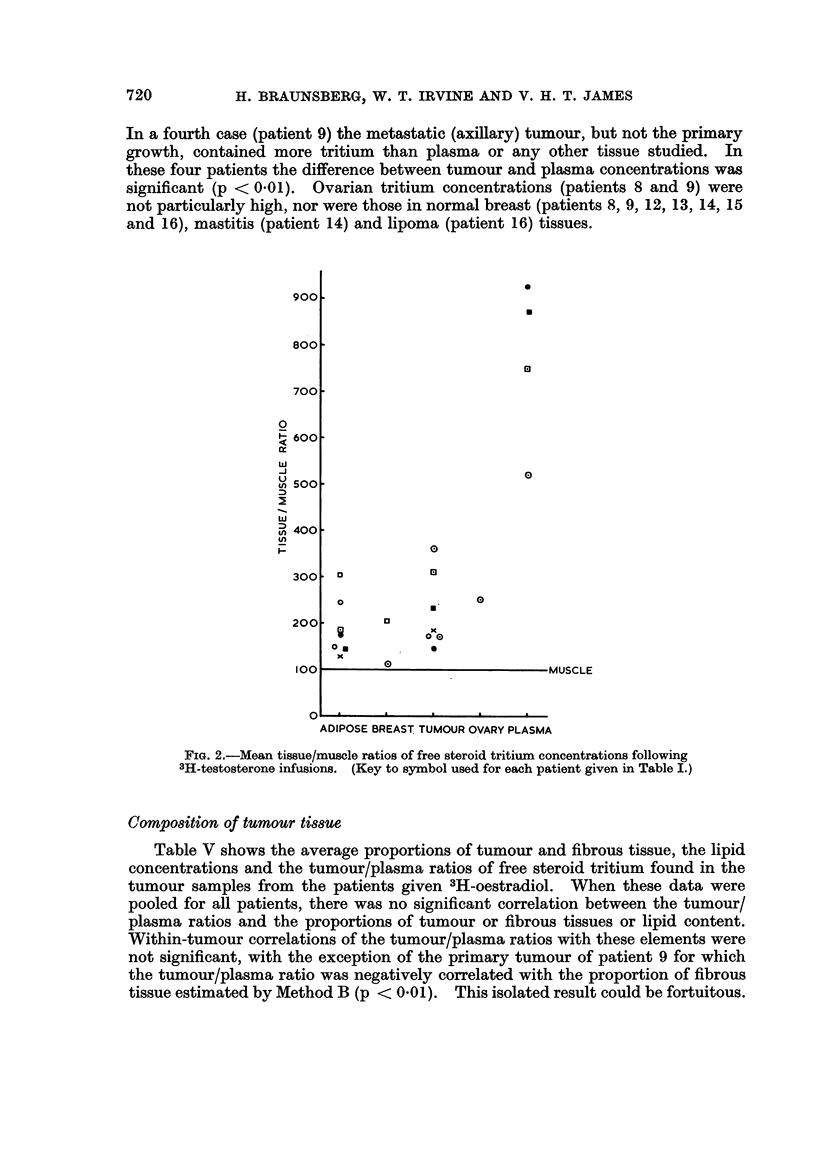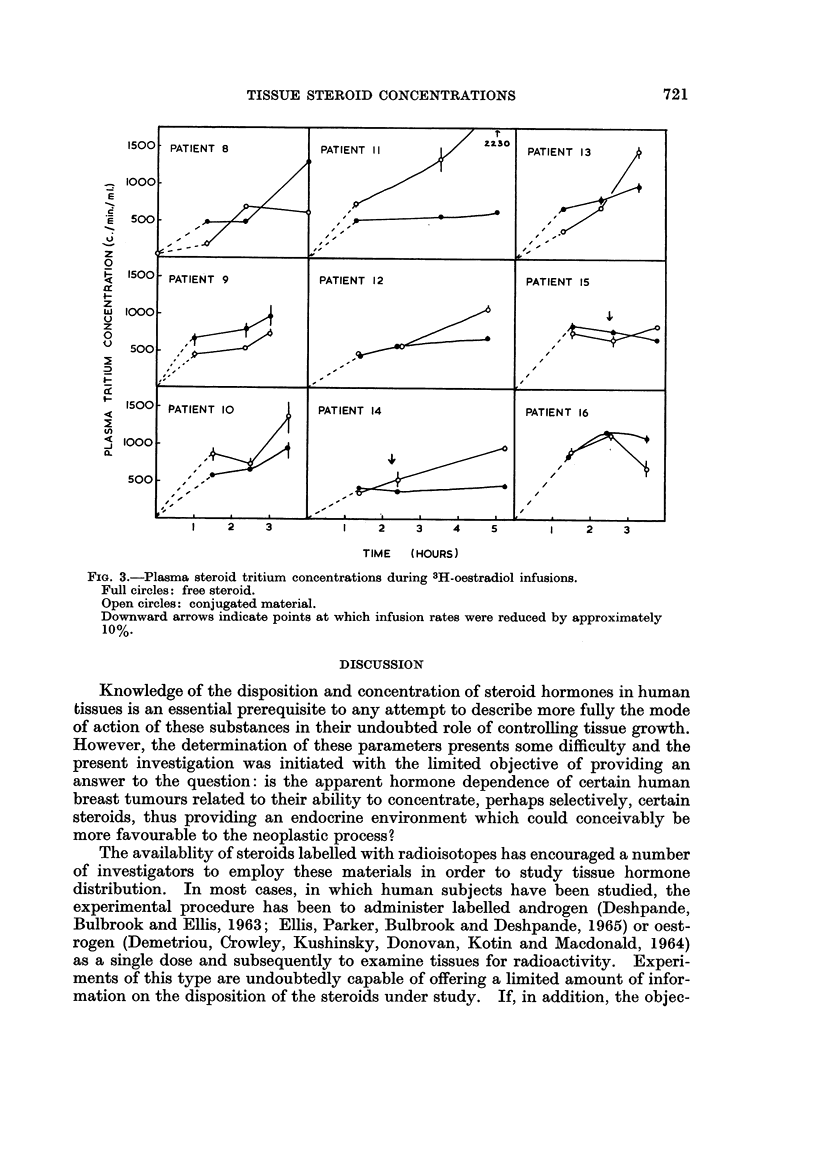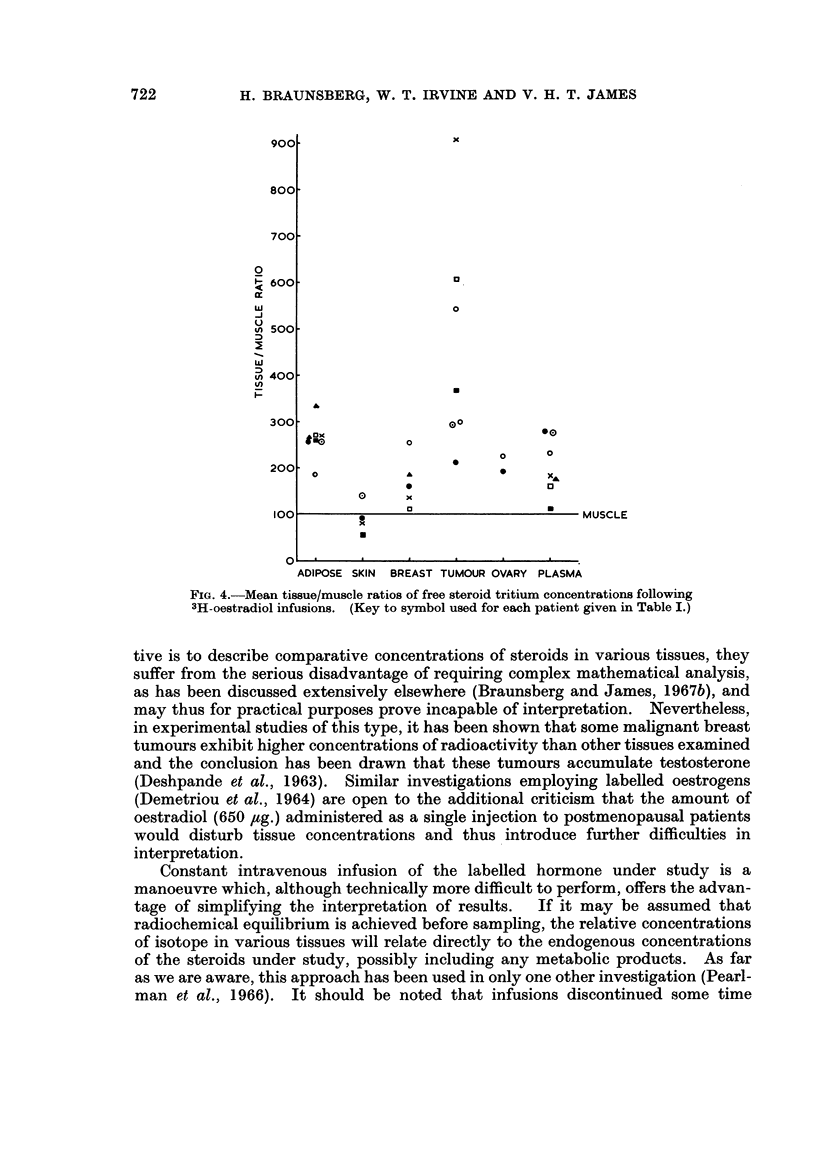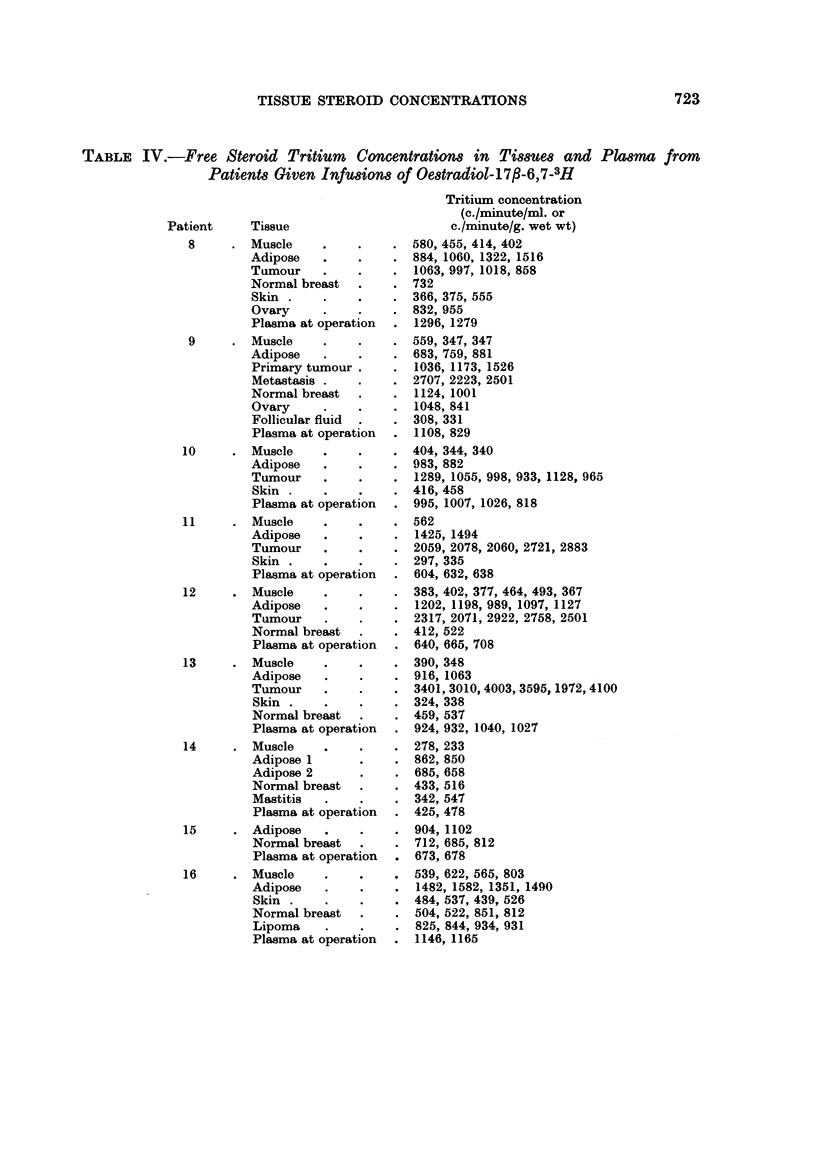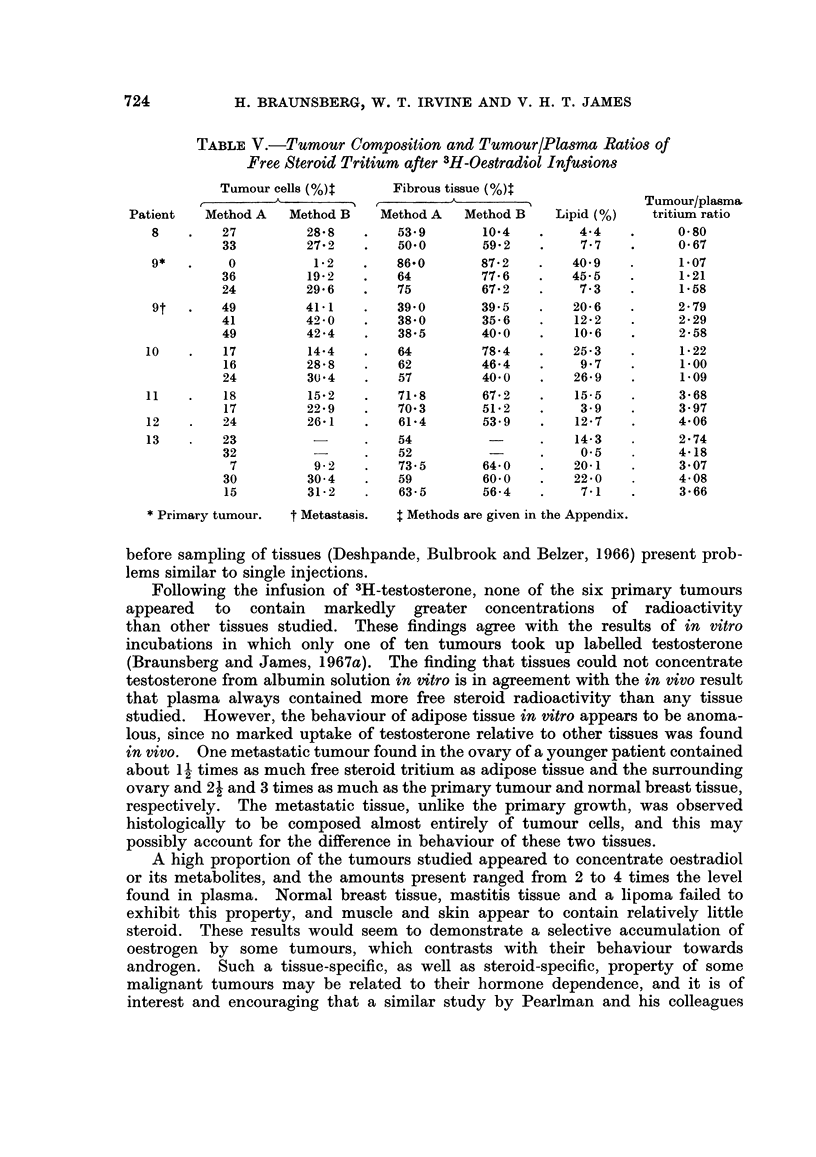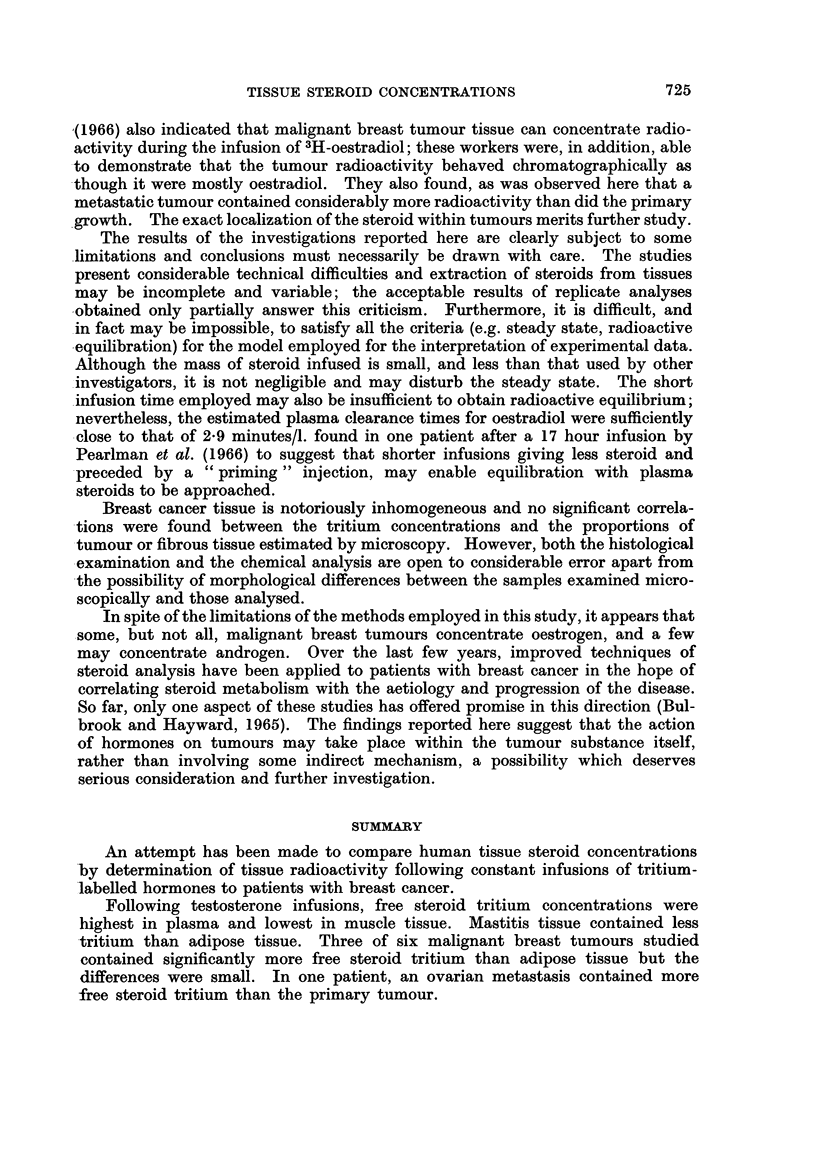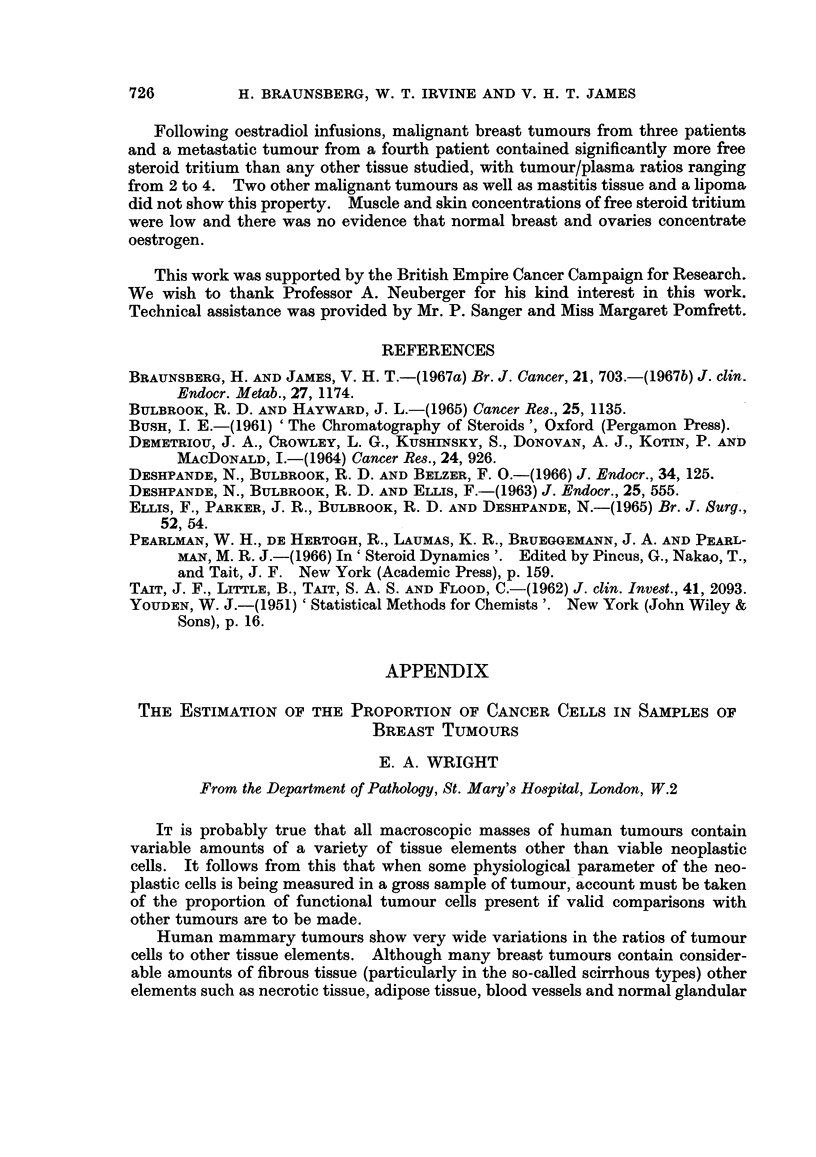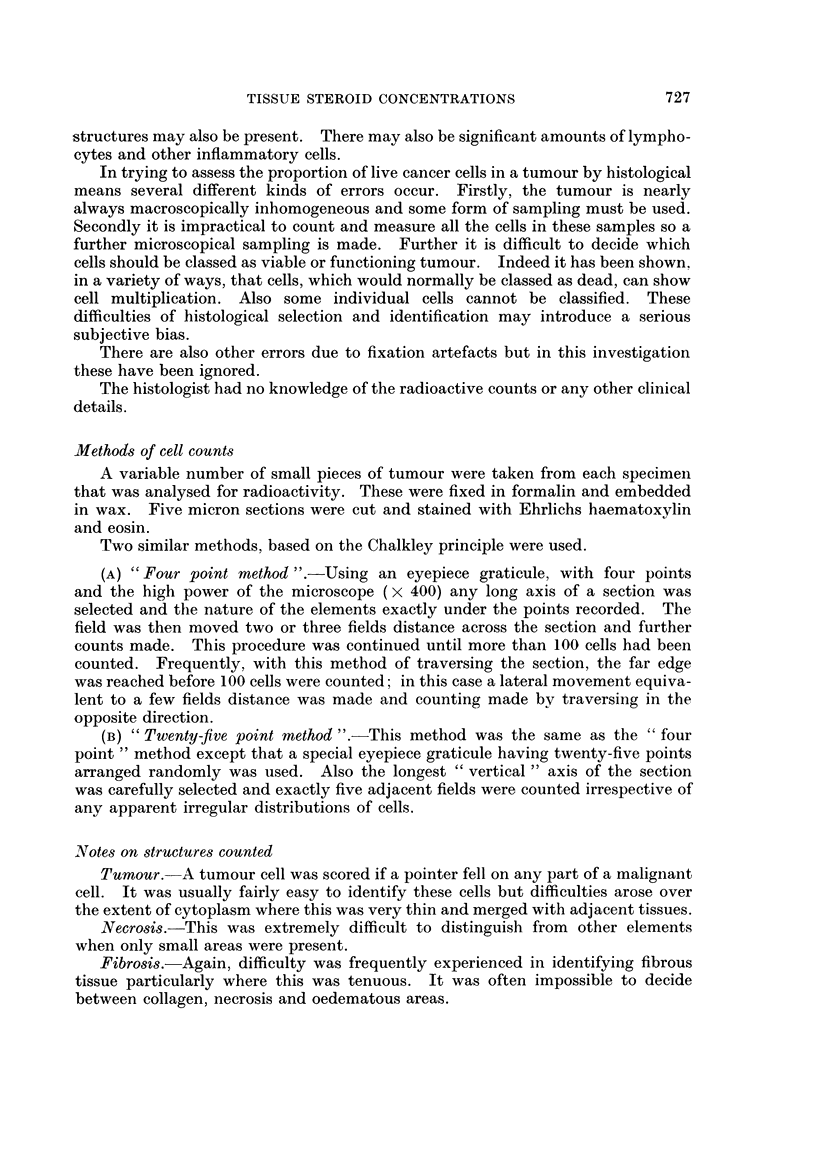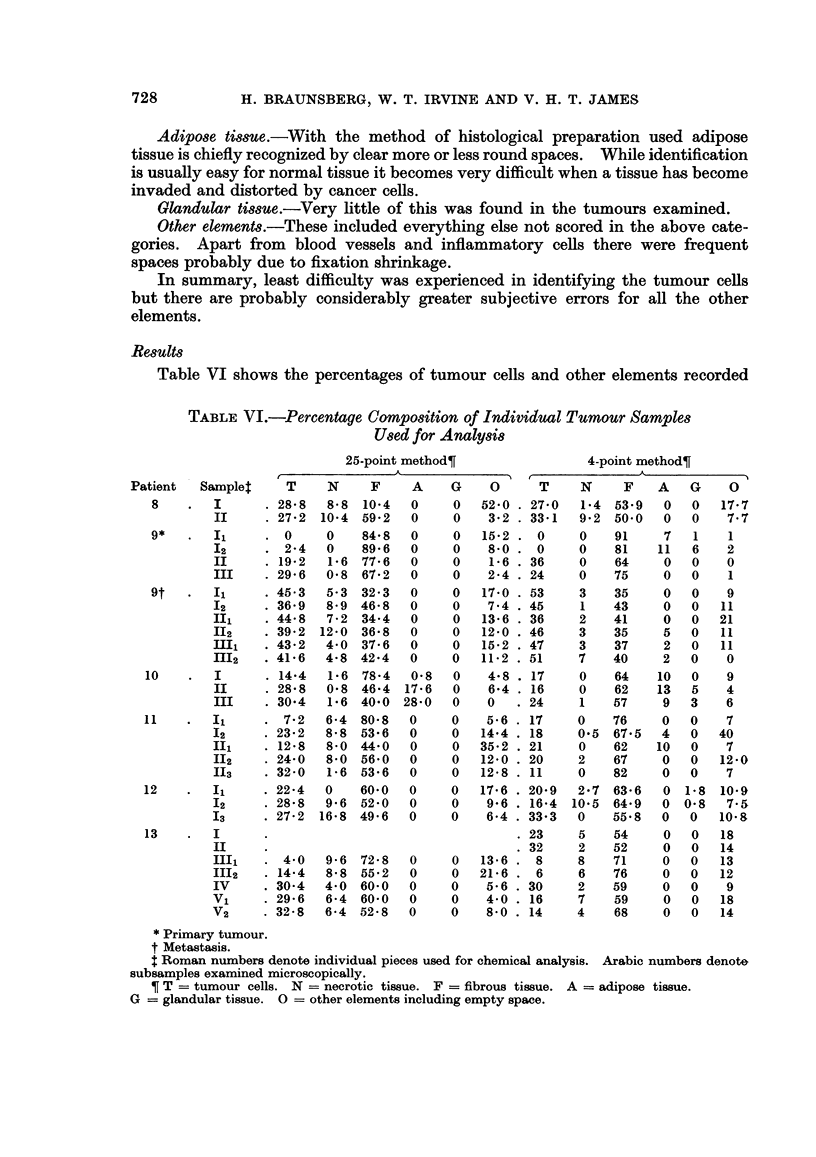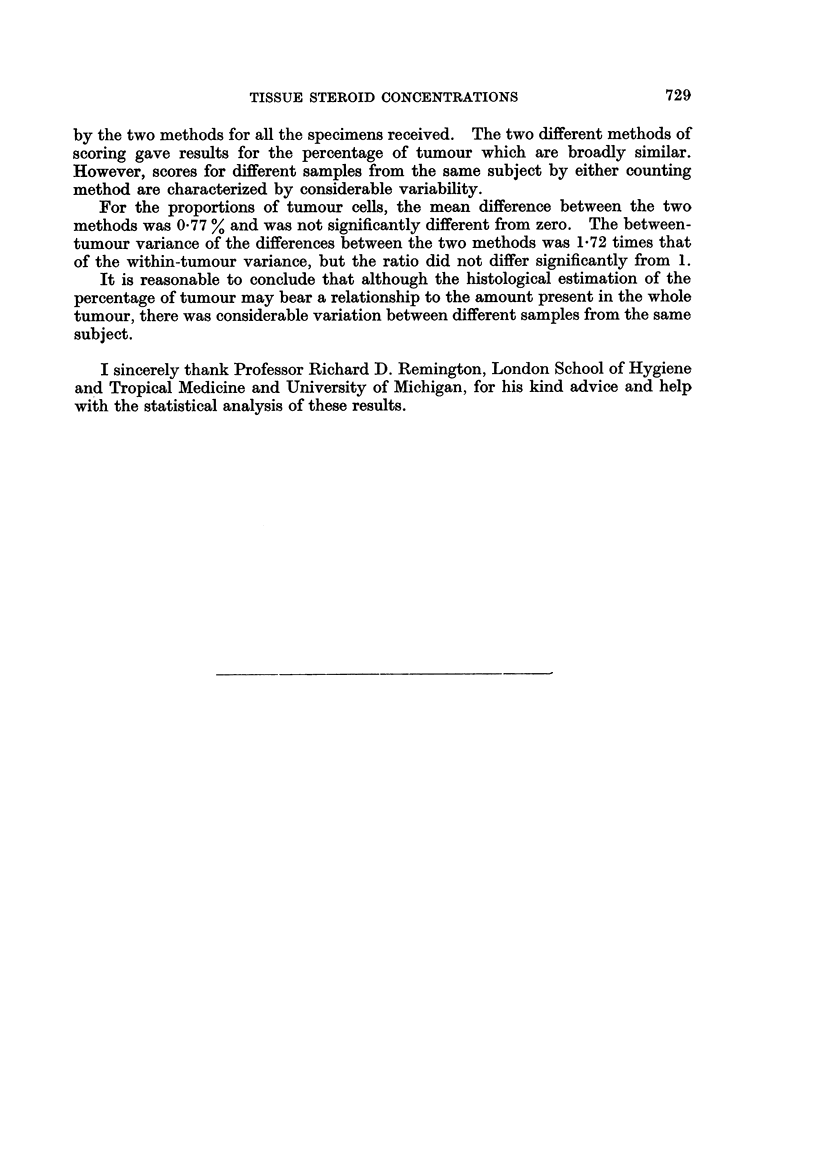# A comparison of steroid hormone concentrations in human tissues including breast cancer.

**DOI:** 10.1038/bjc.1967.84

**Published:** 1967-12

**Authors:** H. Braunsberg, W. T. Irvine, V. H. James


### A COMPARISON OF STEROID HORMONE CONCENTRATIONS IN HUMAN TISSUES INCLUDING BREAST CANCER

HANNELORE BRAUNSBERG, W. T. IRVINE AND V. H. T. JAMES

*From the Department of Chemical Pathology and Surgical Unit, St. Mary's Hospital, London, W.2*

Received for publication July 25, 1967

STUDIES with human tissue slices *in vitro* (Braunsberg and James, 1967*a*) showed that many tissues are capable of binding steroid hormones. Muscle bound relatively little, while adipose tissue showed the largest uptakes of testosterone, oestradiol and progesterone from saline solution. One of ten malignant tumour samples studied took up a relatively large amount of testosterone, but this did not exceed the uptake by adipose tissue. During incubation in serum albumin solution or plasma, little or no steroid was taken up by the tissues studied.

The present investigation was undertaken in order to assess whether *in vitro* equilibration studies can yield meaningful results in relation to tissue hormone concentrations *in vivo* and to compare the distribution of androgen and oestrogen in human tissues with particular reference to malignant breast tumours. To this end, patients with—or suspected of having—breast cancer were given constant infusions of tracer doses of tritium-labelled testosterone or oestradiol in an attempt to reach radioactive equilibrium in the whole body. Under these conditions, the concentrations of free steroid tritium in the tissues are proportional to those of the endogenous hormone and its metabolites. A comparison of individual tissue concentrations in each patient is, therefore, possible. Even when equilibration is incomplete, gross differences in concentrations may be detectable (Braunsberg and James, 1967*b*).

### MATERIALS AND METHODS

*Reagents.*—Petroleum ether (60°–80°) and methylene chloride (both general purpose reagents) were purified as described for hydrocarbons by Bush (1961), except that they were dried over anhydrous sodium sulphate before distillation. Ethanol (Distillers' Company Ltd.) was purified as described for methanol by Bush (1961). Methanol was redistilled and water was glass-distilled. Ether was washed with $\frac{1}{10}$ volume of saturated aqueous ferrous sulphate acidified with hydrochloric acid, followed by water until neutral. It was then dried over anhydrous sodium sulphate and redistilled through a Dufton column. The liquid scintillator was prepared as described in another paper (Braunsberg and James, 1967*a*).

Testosterone-4-$^{14}$C (101 $\mu$Ci/mg.), oestradiol-4-$^{14}$C (116 $\mu$Ci/mg.) and oestradiol-6,7-$^{3}$H (120 $\mu$Ci/$\mu$g.) were from the Radiochemical Centre, Amersham, Bucks. Testosterone-1,2-$^{3}$H (135 $\mu$Ci/$\mu$g.) was kindly provided by the Endocrinology Study Section, National Institutes of Health, Bethesda, Md. The tritium-labelled steroids were stored as dilute solutions (4 to 8 $\mu$Ci/ml.) in dry benzene at $-15°$ C. and were checked for purity by paper chromatography before use and after completion of the last infusion.

*Cleaning of glassware.*—Glassware was cleaned in one of two ways, both of which could be shown to produce negligible count rates when " blanks " were run through the complete analytical procedure following tissue or plasma samples. The glassware was soaked overnight in chromium trioxide dissolved in concentrated nitric acid, rinsed in hot water, agitated in a solution of Pyroneg (Diversey (U.K.) Ltd., Cockfosters, Herts) in an ultrasonic bath, rinsed with hot water and distilled water. Omission of parts of this procedure sometimes led to high blank counts. Alternatively, the glassware was washed in Avion detergent (Diversey (U.K.) Ltd.) in a Hamo glassware washing machine (Camlab (Glass) Ltd., Cambridge) with the maximum number of cycles and rinses, the final rinse being glass-distilled water. Washed glassware was dried in an oven at 112° C.

*Infusions.*—Fifty or 100 $\mu$Ci of steroid was dissolved in 0·1 ml. ethanol and 10 ml. sterile saline (0·9 %) was added. Using a syringe and needle the mixture was slowly injected into a drip bottle containing a freshly prepared 5 to 6·25 % solution of human serum albumin in 400 to 500 ml. sterile saline (0·9 %), while gently shaking the bottle. The albumin reduced the loss of the labelled steroid, due to adsorption, from approximately 80 % to between 20 and 40 %. Immediately after commencement of the infusions, which were given into a cannulated vein, " priming doses " (Tait, Little, Tait and Flood, 1962) of 10 $\mu$Ci steroid, prepared with 0·1 ml. ethanol and 10 ml. saline, were injected into the rubber tubing connecting the cannula to the drip set. The infusions were continued until all tissues had been removed at operation. To ensure constancy, the drip rates were observed throughout the whole procedure, alterations being made by means of a finely adjustable clip (A-C-T Transfusion Regulator, Willen Bros. Ltd., London W.1) when necessary. Timed heparinized blood samples, usually collected at 1½ and 2½ hours after starting the infusion and at the time of removal of the tissues, were centrifuged within 15 minutes and the plasmas stored at −15° C. Tissues collected at operation were placed in a deep freeze within 15 to 30 minutes of removal from the patients and stored at −15° C. until analysed. Table I shows details of the patients studied together with the rates and total times of infusion.

TABLE I.—*Details of Patients Infused with Tritium-Labelled Steroids*

| Patient | Age (years) | Diagnosis | Steroid given | Infusion rate ($\mu$Ci/hour) | Time (hours) | Symbol in Fig. 2 and 4 |
|---|---|---|---|---|---|---|
| 1 | 72 | Carcinoma | Testosterone | 21 | 4.25 | ● |
| 2 | 78 | Carcinoma | Testosterone | 4 | 5 | ○ |
| 3 | 42 | Carcinoma | Testosterone | Not determined | 2·4 | ⊙ |
| 4 | 55 | Carcinoma | Testosterone | 6 | 3·25 | ■ |
| 5 | 55 | Carcinoma | Testosterone | Not determined | 2·5 | × |
| 6 | 73 | Carcinoma* | Testosterone | Not determined | 2·5 | ⊡ |
| 7 | 50 | Mastitis | Testosterone | 5 | 6 | □ |
| 8 | 58 | Carcinoma | Oestradiol | 24 | 4 | ● |
| 9 | 32 | Carcinoma | Oestradiol | Not determined | 3 | ○ |
| 10 | 62 | Carcinoma | Oestradiol | 16·5 | 3·5 | ⊙ |
| 11 | 72 | Carcinoma | Oestradiol | 26 | 3·5 | ■ |
| 12 | 37 | Carcinoma | Oestradiol | Not determined | 4·7 | □ |
| 13 | 65 | Carcinoma | Oestradiol | 18 | 3·25 | × |
| 14 | 39 | Mastitis | Oestradiol | 13 | 5·2 | ▲ |
| 15 | 48 | Cyst | Oestradiol | 22 | 3·3 | |
| 16 | 54 | Lipoma† | Oestradiol | 22·5 | 3·5 | |

* Very necrotic tumour.
† History of carcinoma of other breast.

*Determination of infusion rates.*—The volumes remaining in the drip sets and bottles after the infusions were measured and the total volume received by each patient calculated. Duplicate samples of 1 ml. of infusion mixtures were extracted with 10 ml. methylene chloride and from each extract duplicate aliquots of 0·2 ml. were taken to dryness and counted in 10 ml. scintillator, a minimum of 20,000 counts being accumulated. The tritium concentrations and hence the drip rates, in $\mu$Ci/hr., were calculated.

*Analysis of plasma and tissue samples.*—Plasma (0·25 or 0·5 ml.) was added drop by drop to 50 ml. ether : ethanol (3 : 1) containing 100 counts/min. of the appropriate steroid labelled with $^{14}C$ and agitated in an homogenizer (Measuring and Scientific Equipment Ltd., London S.W.1). The extract was filtered through Whatman No. 1 paper and the extraction repeated twice more with fresh solvent. The volume of the pooled extract was reduced to approximately 10 ml. on a rotatory film evaporator (Wright Scientific Ltd., London, N.W.6). The concentrated extract was transferred to a glass-stoppered test tube, taken to dryness on the evaporator and shaken with 10 ml. 70% aqueous methanol and 10 ml. petroleum ether and centrifuged. The methanol phase was taken to dryness in the evaporator, 4 ml. water added to the residue and the solution extracted 3 times with 10 ml. ether. The pooled ether extract was evaporated to dryness, the residue was dissolved in 10 ml. scintillator and the amounts of $^{14}C$ and $^{3}H$ determined by counting twice for 100 minutes in a Beckman liquid scintillation spectrometer. These samples were found to be free from quenching. The aqueous phase was taken to dryness in the evaporator, the residue of conjugated material was transferred to a counting vial using 3 times 1 ml. ethanol, and 10 ml. scintillator was added. After determination of $^{14}C$ and $^{3}H$ channel count rates, these samples required internal standardization to correct for quenching.

Tissue specimens were freed from other adhering tissues. Wet weights ranging from 0·1 to 1·5 g. were used but recoveries tended to decrease with increase in sample weight. With the exception of muscle and skin, 0·2 to 0·3 g. tissue usually gave count rates which were well above background.

Samples from patients 1, 2 and 3 were pressed through a double stainless steel sieve (mesh 10 and mesh 30) before extraction. However, this method led to some loss of tissue and it could be shown that carefully hand-sliced tissue gave similar results. Tissues from patients 4 to 10 were sliced on a ceramic tile.

Method 1. Weighed tissue preparations were added to 50 ml. ether: ethanol containing 100 counts/minute $^{14}C$-steroid. The extraction was carried out as described for plasma samples except that the petroleum ether washes from the 70% methanol partitions were evaporated and the residues weighed to obtain an estimate of lipid content. This method was used for patients 1 to 10.

Method 2. Tissue samples from patients 11 to 16 were simultaneously ground and extracted with 8 ml. ether : ethanol containing 100 counts/minute $^{14}C$-steroid in a more powerful homogenizer (Silverson Machines Ltd., London, S.E.1). The extraction was repeated twice more with fresh solvent and the pooled extracts treated as described for plasma except that the lipid content was estimated as described for Method 1.

*Calculation of results.*—Correction of $^{14}C$ and $^{3}H$ channel count rates for background, channel overlap and, if necessary, for quenching, calculation of and correction for $^{14}C$ recovery and final calculation of tritium concentrations as counts/minute/ml. plasma or counts/minute/g. tissue were done on an Atlas computer (kindly made available by the Institute of Computer Science, University of London) using a FORTRAN IV programme.

*Histological examination.*—Several small samples of tumour tissue were removed from the pieces used for chemical analysis and examined histologically (see Appendix).

### RESULTS

Plasma and tissue analyses were carried out in batches of six samples. Duplicate reference standards of the $^{14}C$-steroid solution used for addition were pipetted and counted for each batch. Mean $^{14}C$-standard count rates were used to calculate recoveries and the differences between duplicates could be used to estimate the precision of addition from $s = \sqrt{\Sigma d_i^2/2N}$ (Youden, 1951). For 20 pairs of $^{14}C$-testosterone standards the coefficient of variation (s/mean) including counting errors was 4·0 % and for 29 pairs of $^{14}C$-oestradiol standards it was 3·6 %. It is unlikely that errors due to $^{14}C$-recovery correction would exceed 10 %.

Mean recoveries of $^{14}C$-testosterone added to tissue samples were greater than 80 % ($N \geqslant 20$ for each tissue studied) with coefficients of variation ranging from 9·1 % (tumour) to 16·9 % (adipose tissue). Mean recoveries of $^{14}C$-oestradiol added to tissue samples ranged from 70·1 % (plasma) to 82·6 % (tumour) ($N \geqslant 15$ for each tissue) with coefficients of variation ranging from 8·6 % (muscle) to 15·4 % (tumour). Thus the coefficients of variation were considerably greater than those expected for pipetting and counting errors only, and it was, therefore, necessary to correct tissue tritium concentrations for losses in each case.

Frozen plasma samples, allowed to reach room temperature, always contained a precipitate, probably of denatured protein, which made pipetting difficult. Plasma from a patient given $^{3}H$-oestradiol gave results of 879 and 867 counts/minute/ml. before, and 906 and 883 counts/minute/ml. after centrifuging. The precipitate, therefore, did not appear to carry down the steroid and could be removed before pipetting. Plasma samples from patients given $^{3}H$-testosterone or $^{3}H$-oestradiol could be kept at room temperature for 4 to 5 hours without showing any significant change in free steroid tritium concentration.

Table II shows the results of experiments in which parallel tissue samples were analysed by Methods 1 and 2. Recoveries of $^{14}C$-steroid and tissue tritium concentrations were similar in most cases, but in order to avoid any small differences, tissues from any one patient were anlysed by one method only. Method 2 is more convenient than Method 1.

*Testosterone infusions.*—Plasma curves for four of the seven patients studied are shown in Fig. 1. In three patients, radioactive equilibration of the plasma free steroid appeared to be approached after approximately 2 hours, but in patient 4 the curve was still rising steeply after 3 hours. Equilibration with the conjugated material was slower than that with the free steroid. The estimated plasma clearance times (cf. Pearlman, de Hertogh, Laumas, Brueggemann and Pearlman, 1966) for patients 1 and 4 were 6 and 27 minutes/l., respectively.

The results for tissues are shown in Table III together with those for plasma taken at operation, and mean concentrations are plotted in Fig. 2 as tissue/muscle ratios (muscle = 100). Free steroid tritium concentrations were highest in plasma and lowest in muscle. The results for adipose tissue were significantly higher than muscle in 5 of 7 cases ($p < 0·01$), and in 3 of the 6 patients with cancer the breast tumour tissue contained slightly more steroid tritium than adipose tissue

TABLE II.—*Comparison of Two Methods for Determining Free Steroid Tritium in Tissues*

| Tissue | Sample | Tritium Concentration (c./minute/g. wet wt.) Method 1* | Method 2† | $^{14}C$ Recovery (%) Method 1* | Method 2† |
|---|---|---|---|---|---|
| Muscle | 1 | 493 | 383 | 84·7 | 78·3 |
| | 2 | 367 | 402, 377, 464 | 80·6 | 76·6, 76·8, 57·7 |
| | 3 | 622 | 539 | 78·0 | 79·4 |
| | 4 | 803 | 565 | 74·9 | 85·0 |
| Adipose | 1 | 1202 | 989 | 66·6 | 66·8 |
| | 2 | 1198 | 1097 | 66·1 | 78·8 |
| | 3 | 1582 | 1482 | 65·8 | 76·4 |
| | 4 | 1490 | 1351 | 85·5 | 69·5 |
| Skin | 1 | 537 | 484 | 73·9 | 63·3 |
| | 2 | 526 | 439 | 79·9 | 57·2 |
| Breast | 1 | 522 | 504 | 82·3 | 91·5 |
| | 2 | 812 | 851 | 82·7 | 90·7 |
| Tumour | | 2922 | 2501 | 82·8 | 81·3 |
| Lipoma | 1 | 844 | 825 | 75·0 | 86·9 |
| | 2 | 931 | 934 | 59·9 | 83·4 |

* Method 1: hand-sliced, extracted in MSE homogeniser (used for patients 4 to 10).

† Method 2: minced and extracted simultaneously in Silverson homogeniser (used for patients 11 to 16).

FIG. 1.—Plasma steroid tritium concentrations during $^{3}H$-testosterone infusions. Full circles: free steroid. Open circles: conjugated material.

TABLE III.—*Free Steroid Tritium Concentrations in Tissues and Plasma from Patients Given Infusions of Testosterone*-1,2-$^3H$

| Patient | Tissue | Tritium concentration (c./minute/ml. or c./minute/g. wet wt) |
|---|---|---|
| 1 | Muscle | 334, 231, 281, 210 |
| | Adipose | 439, 432, 482 |
| | Tumour | 360, 411, 336 |
| | Plasma at operation | 2490, 2392 |
| 2 | Muscle | 147, 149 |
| | Adipose | 232, 209, 223 |
| | Tumour | 283, 231, 253 |
| 3 | Muscle | 389, 376 |
| | Adipose | 956, 885, 977 |
| | Primary tumour | 638, 671, 664 |
| | Metastasis | 1359, 1415 |
| | Normal breast | 461, 411 |
| | Ovary | 965 |
| | Plasma at operation | 1985, 1978 |
| 4 | Muscle | 335, 358 |
| | Adipose | 813, 505, 577, 426 |
| | Tumour | 803, 800 |
| | Plasma at operation | 3104, 2974, 3080, 2933 |
| 5 | Muscle | 104, 111, 135, 218 |
| | Adipose | 160, 167, 206, 190 |
| | Tumour | 292, 254, 273, 232 |
| 6 | Muscle | 245, 243, 273, 295 |
| | Adipose | 437, 526 |
| | Tumour | 619, 955, 835, 878 |
| | Plasma at operation | 2117, 1841 |
| 7 | Muscle | 131, 142 |
| | Adipose | 460, 569, 337, 362, 337 |
| | Mastitis | 238, 284, 274, 318 |

($p < 0{\cdot}05$, $p < 0{\cdot}02$ and $p < 0{\cdot}002$ respectively). The metastic tumour (found in the ovary) of patient 3 contained twice as much free steroid tritium as the primary tumour and somewhat more than the surrounding ovarian tissue. Normal breast tissue from this patient contained slightly less tritium than the primary tumour. Mastitis tissue (patient 7) contained less tritium than adipose tissue.

*Oestradiol infusions*

The plasma curves are shown in Fig. 3. Equilibration with the free steroid was approached after $1\frac{1}{2}$ to 2 hours. Plasma tritium concentrations were lower in these patients than in those given $^3$H-testosterone. This is obvious from a comparison of patient 1 (Fig. 1) with patients 8, 11, 15 and 16 (Fig. 3) who were given infusions at similar rates. The rise in free steroid tritium concentration seen in some of the samples taken at operation was too large to be explained by blood loss. The estimated plasma clearance times (cf. Pearlman *et al.*, 1966) for patients 8, 10, 11, 13, 14, 15 and 16 were 2·9, 3·0, 1·3, 3·0, 1·9, 1·6 and 2·8 minutes/l., respectively.

Table IV shows the results for tissues and plasmas taken at operation and mean tissue/muscle ratios are plotted in Fig. 4. In 3 of the 6 patients with cancer, the tumour tissue contained by far the highest concentrations of free steroid tritium.

In a fourth case (patient 9) the metastatic (axillary) tumour, but not the primary growth, contained more tritium than plasma or any other tissue studied. In these four patients the difference between tumour and plasma concentrations was significant ($p < 0{\cdot}01$). Ovarian tritium concentrations (patients 8 and 9) were not particularly high, nor were those in normal breast (patients 8, 9, 12, 13, 14, 15 and 16), mastitis (patient 14) and lipoma (patient 16) tissues.

FIG. 2.—Mean tissue/muscle ratios of free steroid tritium concentrations following $^{3}$H-testosterone infusions. (Key to symbol used for each patient given in Table I.)

*Composition of tumour tissue*

Table V shows the average proportions of tumour and fibrous tissue, the lipid concentrations and the tumour/plasma ratios of free steroid tritium found in the tumour samples from the patients given $^{3}$H-oestradiol. When these data were pooled for all patients, there was no significant correlation between the tumour/plasma ratios and the proportions of tumour or fibrous tissues or lipid content. Within-tumour correlations of the tumour/plasma ratios with these elements were not significant, with the exception of the primary tumour of patient 9 for which the tumour/plasma ratio was negatively correlated with the proportion of fibrous tissue estimated by Method B ($p < 0{\cdot}01$). This isolated result could be fortuitous.

FIG. 3.—Plasma steroid tritium concentrations during $^{3}H$-oestradiol infusions.
Full circles: free steroid.
Open circles: conjugated material.
Downward arrows indicate points at which infusion rates were reduced by approximately 10%.

### DISCUSSION

Knowledge of the disposition and concentration of steroid hormones in human tissues is an essential prerequisite to any attempt to describe more fully the mode of action of these substances in their undoubted role of controlling tissue growth. However, the determination of these parameters presents some difficulty and the present investigation was initiated with the limited objective of providing an answer to the question: is the apparent hormone dependence of certain human breast tumours related to their ability to concentrate, perhaps selectively, certain steroids, thus providing an endocrine environment which could conceivably be more favourable to the neoplastic process?

The availablity of steroids labelled with radioisotopes has encouraged a number of investigators to employ these materials in order to study tissue hormone distribution. In most cases, in which human subjects have been studied, the experimental procedure has been to administer labelled androgen (Deshpande, Bulbrook and Ellis, 1963; Ellis, Parker, Bulbrook and Deshpande, 1965) or oestrogen (Demetriou, Crowley, Kushinsky, Donovan, Kotin and Macdonald, 1964) as a single dose and subsequently to examine tissues for radioactivity. Experiments of this type are undoubtedly capable of offering a limited amount of information on the disposition of the steroids under study. If, in addition, the objec-

FIG. 4.—Mean tissue/muscle ratios of free steroid tritium concentrations following $^3$H-oestradiol infusions. (Key to symbol used for each patient given in Table I.)

tive is to describe comparative concentrations of steroids in various tissues, they suffer from the serious disadvantage of requiring complex mathematical analysis, as has been discussed extensively elsewhere (Braunsberg and James, 1967*b*), and may thus for practical purposes prove incapable of interpretation. Nevertheless, in experimental studies of this type, it has been shown that some malignant breast tumours exhibit higher concentrations of radioactivity than other tissues examined and the conclusion has been drawn that these tumours accumulate testosterone (Deshpande *et al.*, 1963). Similar investigations employing labelled oestrogens (Demetriou *et al.*, 1964) are open to the additional criticism that the amount of oestradiol (650 $\mu$g.) administered as a single injection to postmenopausal patients would disturb tissue concentrations and thus introduce further difficulties in interpretation.

Constant intravenous infusion of the labelled hormone under study is a manoeuvre which, although technically more difficult to perform, offers the advantage of simplifying the interpretation of results. If it may be assumed that radiochemical equilibrium is achieved before sampling, the relative concentrations of isotope in various tissues will relate directly to the endogenous concentrations of the steroids under study, possibly including any metabolic products. As far as we are aware, this approach has been used in only one other investigation (Pearlman *et al.*, 1966). It should be noted that infusions discontinued some time

TABLE IV.—*Free Steroid Tritium Concentrations in Tissues and Plasma from Patients Given Infusions of Oestradiol*-17β-6,7-$^{3}$*H*

| Patient | Tissue | Tritium concentration (c./minute/ml. or c./minute/g. wet wt) |
|---|---|---|
| 8 | Muscle | 580, 455, 414, 402 |
| | Adipose | 884, 1060, 1322, 1516 |
| | Tumour | 1063, 997, 1018, 858 |
| | Normal breast | 732 |
| | Skin | 366, 375, 555 |
| | Ovary | 832, 955 |
| | Plasma at operation | 1296, 1279 |
| 9 | Muscle | 559, 347, 347 |
| | Adipose | 683, 759, 881 |
| | Primary tumour | 1036, 1173, 1526 |
| | Metastasis | 2707, 2223, 2501 |
| | Normal breast | 1124, 1001 |
| | Ovary | 1048, 841 |
| | Follicular fluid | 308, 331 |
| | Plasma at operation | 1108, 829 |
| 10 | Muscle | 404, 344, 340 |
| | Adipose | 983, 882 |
| | Tumour | 1289, 1055, 998, 933, 1128, 965 |
| | Skin | 416, 458 |
| | Plasma at operation | 995, 1007, 1026, 818 |
| 11 | Muscle | 562 |
| | Adipose | 1425, 1494 |
| | Tumour | 2059, 2078, 2060, 2721, 2883 |
| | Skin | 297, 335 |
| | Plasma at operation | 604, 632, 638 |
| 12 | Muscle | 383, 402, 377, 464, 493, 367 |
| | Adipose | 1202, 1198, 989, 1097, 1127 |
| | Tumour | 2317, 2071, 2922, 2758, 2501 |
| | Normal breast | 412, 522 |
| | Plasma at operation | 640, 665, 708 |
| 13 | Muscle | 390, 348 |
| | Adipose | 916, 1063 |
| | Tumour | 3401, 3010, 4003, 3595, 1972, 4100 |
| | Skin | 324, 338 |
| | Normal breast | 459, 537 |
| | Plasma at operation | 924, 932, 1040, 1027 |
| 14 | Muscle | 278, 233 |
| | Adipose 1 | 862, 850 |
| | Adipose 2 | 685, 658 |
| | Normal breast | 433, 516 |
| | Mastitis | 342, 547 |
| | Plasma at operation | 425, 478 |
| 15 | Adipose | 904, 1102 |
| | Normal breast | 712, 685, 812 |
| | Plasma at operation | 673, 678 |
| 16 | Muscle | 539, 622, 565, 803 |
| | Adipose | 1482, 1582, 1351, 1490 |
| | Skin | 484, 537, 439, 526 |
| | Normal breast | 504, 522, 851, 812 |
| | Lipoma | 825, 844, 934, 931 |
| | Plasma at operation | 1146, 1165 |

TABLE V.—*Tumour Composition and Tumour/Plasma Ratios of Free Steroid Tritium after $^3$H-Oestradiol Infusions*

| Patient | Tumour cells (%)‡ Method A | Tumour cells (%)‡ Method B | Fibrous tissue (%)‡ Method A | Fibrous tissue (%)‡ Method B | Lipid (%) | Tumour/plasma tritium ratio |
|---|---|---|---|---|---|---|
| 8 | 27 | 28·8 | 53·9 | 10·4 | 4·4 | 0·80 |
| | 33 | 27·2 | 50·0 | 59·2 | 7·7 | 0·67 |
| 9* | 0 | 1·2 | 86·0 | 87·2 | 40·9 | 1·07 |
| | 36 | 19·2 | 64 | 77·6 | 45·5 | 1·21 |
| | 24 | 29·6 | 75 | 67·2 | 7·3 | 1·58 |
| 9† | 49 | 41·1 | 39·0 | 39·5 | 20·6 | 2·79 |
| | 41 | 42·0 | 38·0 | 35·6 | 12·2 | 2·29 |
| | 49 | 42·4 | 38·5 | 40·0 | 10·6 | 2·58 |
| 10 | 17 | 14·4 | 64 | 78·4 | 25·3 | 1·22 |
| | 16 | 28·8 | 62 | 46·4 | 9·7 | 1·00 |
| | 24 | 30·4 | 57 | 40·0 | 26·9 | 1·09 |
| 11 | 18 | 15·2 | 71·8 | 67·2 | 15·5 | 3·68 |
| | 17 | 22·9 | 70·3 | 51·2 | 3·9 | 3·97 |
| 12 | 24 | 26·1 | 61·4 | 53·9 | 12·7 | 4·06 |
| 13 | 23 | — | 54 | — | 14·3 | 2·74 |
| | 32 | — | 52 | — | 0·5 | 4·18 |
| | 7 | 9·2 | 73·5 | 64·0 | 20·1 | 3·07 |
| | 30 | 30·4 | 59 | 60·0 | 22·0 | 4·08 |
| | 15 | 31·2 | 63·5 | 56·4 | 7·1 | 3·66 |

* Primary tumour. † Metastasis. ‡ Methods are given in the Appendix.

before sampling of tissues (Deshpande, Bulbrook and Belzer, 1966) present problems similar to single injections.

Following the infusion of $^3$H-testosterone, none of the six primary tumours appeared to contain markedly greater concentrations of radioactivity than other tissues studied. These findings agree with the results of *in vitro* incubations in which only one of ten tumours took up labelled testosterone (Braunsberg and James, 1967*a*). The finding that tissues could not concentrate testosterone from albumin solution *in vitro* is in agreement with the *in vivo* result that plasma always contained more free steroid radioactivity than any tissue studied. However, the behaviour of adipose tissue *in vitro* appears to be anomalous, since no marked uptake of testosterone relative to other tissues was found *in vivo*. One metastatic tumour found in the ovary of a younger patient contained about 1½ times as much free steroid tritium as adipose tissue and the surrounding ovary and 2½ and 3 times as much as the primary tumour and normal breast tissue, respectively. The metastatic tissue, unlike the primary growth, was observed histologically to be composed almost entirely of tumour cells, and this may possibly account for the difference in behaviour of these two tissues.

A high proportion of the tumours studied appeared to concentrate oestradiol or its metabolites, and the amounts present ranged from 2 to 4 times the level found in plasma. Normal breast tissue, mastitis tissue and a lipoma failed to exhibit this property, and muscle and skin appear to contain relatively little steroid. These results would seem to demonstrate a selective accumulation of oestrogen by some tumours, which contrasts with their behaviour towards androgen. Such a tissue-specific, as well as steroid-specific, property of some malignant tumours may be related to their hormone dependence, and it is of interest and encouraging that a similar study by Pearlman and his colleagues

(1966) also indicated that malignant breast tumour tissue can concentrate radioactivity during the infusion of $^3$H-oestradiol; these workers were, in addition, able to demonstrate that the tumour radioactivity behaved chromatographically as though it were mostly oestradiol. They also found, as was observed here that a metastatic tumour contained considerably more radioactivity than did the primary growth. The exact localization of the steroid within tumours merits further study.

The results of the investigations reported here are clearly subject to some limitations and conclusions must necessarily be drawn with care. The studies present considerable technical difficulties and extraction of steroids from tissues may be incomplete and variable; the acceptable results of replicate analyses obtained only partially answer this criticism. Furthermore, it is difficult, and in fact may be impossible, to satisfy all the criteria (e.g. steady state, radioactive equilibration) for the model employed for the interpretation of experimental data. Although the mass of steroid infused is small, and less than that used by other investigators, it is not negligible and may disturb the steady state. The short infusion time employed may also be insufficient to obtain radioactive equilibrium; nevertheless, the estimated plasma clearance times for oestradiol were sufficiently close to that of 2·9 minutes/l. found in one patient after a 17 hour infusion by Pearlman *et al.* (1966) to suggest that shorter infusions giving less steroid and preceded by a " priming " injection, may enable equilibration with plasma steroids to be approached.

Breast cancer tissue is notoriously inhomogeneous and no significant correlations were found between the tritium concentrations and the proportions of tumour or fibrous tissue estimated by microscopy. However, both the histological examination and the chemical analysis are open to considerable error apart from the possibility of morphological differences between the samples examined microscopically and those analysed.

In spite of the limitations of the methods employed in this study, it appears that some, but not all, malignant breast tumours concentrate oestrogen, and a few may concentrate androgen. Over the last few years, improved techniques of steroid analysis have been applied to patients with breast cancer in the hope of correlating steroid metabolism with the aetiology and progression of the disease. So far, only one aspect of these studies has offered promise in this direction (Bulbrook and Hayward, 1965). The findings reported here suggest that the action of hormones on tumours may take place within the tumour substance itself, rather than involving some indirect mechanism, a possibility which deserves serious consideration and further investigation.

### SUMMARY

An attempt has been made to compare human tissue steroid concentrations by determination of tissue radioactivity following constant infusions of tritium-labelled hormones to patients with breast cancer.

Following testosterone infusions, free steroid tritium concentrations were highest in plasma and lowest in muscle tissue. Mastitis tissue contained less tritium than adipose tissue. Three of six malignant breast tumours studied contained significantly more free steroid tritium than adipose tissue but the differences were small. In one patient, an ovarian metastasis contained more free steroid tritium than the primary tumour.

Following oestradiol infusions, malignant breast tumours from three patients and a metastatic tumour from a fourth patient contained significantly more free steroid tritium than any other tissue studied, with tumour/plasma ratios ranging from 2 to 4. Two other malignant tumours as well as mastitis tissue and a lipoma did not show this property. Muscle and skin concentrations of free steroid tritium were low and there was no evidence that normal breast and ovaries concentrate oestrogen.

This work was supported by the British Empire Cancer Campaign for Research. We wish to thank Professor A. Neuberger for his kind interest in this work. Technical assistance was provided by Mr. P. Sanger and Miss Margaret Pomfrett.

### REFERENCES

Braunsberg, H. and James, V. H. T.—(1967*a*) *Br. J. Cancer*, **21**, 703.—(1967*b*) *J. clin. Endocr. Metab.*, **27**, 1174.
Bulbrook, R. D. and Hayward, J. L.—(1965) *Cancer Res.*, **25**, 1135.
Bush, I. E.—(1961) ' The Chromatography of Steroids ', Oxford (Pergamon Press).
Demetriou, J. A., Crowley, L. G., Kushinsky, S., Donovan, A. J., Kotin, P. and MacDonald, I.—(1964) *Cancer Res.*, **24**, 926.
Deshpande, N., Bulbrook, R. D. and Belzer, F. O.—(1966) *J. Endocr.*, **34**, 125.
Deshpande, N., Bulbrook, R. D. and Ellis, F.—(1963) *J. Endocr.*, **25**, 555.
Ellis, F., Parker, J. R., Bulbrook, R. D. and Deshpande, N.—(1965) *Br. J. Surg.*, **52**, 54.
Pearlman, W. H., de Hertogh, R., Laumas, K. R., Brueggemann, J. A. and Pearlman, M. R. J.—(1966) In ' Steroid Dynamics '. Edited by Pincus, G., Nakao, T., and Tait, J. F. New York (Academic Press), p. 159.
Tait, J. F., Little, B., Tait, S. A. S. and Flood, C.—(1962) *J. clin. Invest.*, **41**, 2093.
Youden, W. J.—(1951) ' Statistical Methods for Chemists '. New York (John Wiley & Sons), p. 16.

### APPENDIX

### The Estimation of the Proportion of Cancer Cells in Samples of Breast Tumours

E. A. WRIGHT

*From the Department of Pathology, St. Mary's Hospital, London, W.2*

It is probably true that all macroscopic masses of human tumours contain variable amounts of a variety of tissue elements other than viable neoplastic cells. It follows from this that when some physiological parameter of the neoplastic cells is being measured in a gross sample of tumour, account must be taken of the proportion of functional tumour cells present if valid comparisons with other tumours are to be made.

Human mammary tumours show very wide variations in the ratios of tumour cells to other tissue elements. Although many breast tumours contain considerable amounts of fibrous tissue (particularly in the so-called scirrhous types) other elements such as necrotic tissue, adipose tissue, blood vessels and normal glandular structures may also be present. There may also be significant amounts of lymphocytes and other inflammatory cells.

In trying to assess the proportion of live cancer cells in a tumour by histological means several different kinds of errors occur. Firstly, the tumour is nearly always macroscopically inhomogeneous and some form of sampling must be used. Secondly it is impractical to count and measure all the cells in these samples so a further microscopical sampling is made. Further it is difficult to decide which cells should be classed as viable or functioning tumour. Indeed it has been shown, in a variety of ways, that cells, which would normally be classed as dead, can show cell multiplication. Also some individual cells cannot be classified. These difficulties of histological selection and identification may introduce a serious subjective bias.

There are also other errors due to fixation artefacts but in this investigation these have been ignored.

The histologist had no knowledge of the radioactive counts or any other clinical details.

### *Methods of cell counts*

A variable number of small pieces of tumour were taken from each specimen that was analysed for radioactivity. These were fixed in formalin and embedded in wax. Five micron sections were cut and stained with Ehrlichs haematoxylin and eosin.

Two similar methods, based on the Chalkley principle were used.

(A) "*Four point method*".—Using an eyepiece graticule, with four points and the high power of the microscope ($\times$ 400) any long axis of a section was selected and the nature of the elements exactly under the points recorded. The field was then moved two or three fields distance across the section and further counts made. This procedure was continued until more than 100 cells had been counted. Frequently, with this method of traversing the section, the far edge was reached before 100 cells were counted; in this case a lateral movement equivalent to a few fields distance was made and counting made by traversing in the opposite direction.

(B) "*Twenty-five point method*".—This method was the same as the "four point" method except that a special eyepiece graticule having twenty-five points arranged randomly was used. Also the longest "vertical" axis of the section was carefully selected and exactly five adjacent fields were counted irrespective of any apparent irregular distributions of cells.

### *Notes on structures counted*

*Tumour.*—A tumour cell was scored if a pointer fell on any part of a malignant cell. It was usually fairly easy to identify these cells but difficulties arose over the extent of cytoplasm where this was very thin and merged with adjacent tissues.

*Necrosis.*—This was extremely difficult to distinguish from other elements when only small areas were present.

*Fibrosis.*—Again, difficulty was frequently experienced in identifying fibrous tissue particularly where this was tenuous. It was often impossible to decide between collagen, necrosis and oedematous areas.

*Adipose tissue.*—With the method of histological preparation used adipose tissue is chiefly recognized by clear more or less round spaces. While identification is usually easy for normal tissue it becomes very difficult when a tissue has become invaded and distorted by cancer cells.

*Glandular tissue.*—Very little of this was found in the tumours examined.

*Other elements.*—These included everything else not scored in the above categories. Apart from blood vessels and inflammatory cells there were frequent spaces probably due to fixation shrinkage.

In summary, least difficulty was experienced in identifying the tumour cells but there are probably considerably greater subjective errors for all the other elements.

### *Results*

Table VI shows the percentages of tumour cells and other elements recorded

TABLE VI.—*Percentage Composition of Individual Tumour Samples Used for Analysis*

| | | 25-point method¶ | | | | | | 4-point method¶ | | | | | |
|---|---|---|---|---|---|---|---|---|---|---|---|---|---|
| Patient | Sample‡ | T | N | F | A | G | O | T | N | F | A | G | O |
| 8 | I | 28·8 | 8·8 | 10·4 | 0 | 0 | 52·0 | 27·0 | 1·4 | 53·9 | 0 | 0 | 17·7 |
| | II | 27·2 | 10·4 | 59·2 | 0 | 0 | 3·2 | 33·1 | 9·2 | 50·0 | 0 | 0 | 7·7 |
| 9* | $I_1$ | 0 | 0 | 84·8 | 0 | 0 | 15·2 | 0 | 0 | 91 | 7 | 1 | 1 |
| | $I_2$ | 2·4 | 0 | 89·6 | 0 | 0 | 8·0 | 0 | 0 | 81 | 11 | 6 | 2 |
| | II | 19·2 | 1·6 | 77·6 | 0 | 0 | 1·6 | 36 | 0 | 64 | 0 | 0 | 0 |
| | III | 29·6 | 0·8 | 67·2 | 0 | 0 | 2·4 | 24 | 0 | 75 | 0 | 0 | 1 |
| 9† | $I_1$ | 45·3 | 5·3 | 32·3 | 0 | 0 | 17·0 | 53 | 3 | 35 | 0 | 0 | 9 |
| | $I_2$ | 36·9 | 8·9 | 46·8 | 0 | 0 | 7·4 | 45 | 1 | 43 | 0 | 0 | 11 |
| | $II_1$ | 44·8 | 7·2 | 34·4 | 0 | 0 | 13·6 | 36 | 2 | 41 | 0 | 0 | 21 |
| | $II_2$ | 39·2 | 12·0 | 36·8 | 0 | 0 | 12·0 | 46 | 3 | 35 | 5 | 0 | 11 |
| | $III_1$ | 43·2 | 4·0 | 37·6 | 0 | 0 | 15·2 | 47 | 3 | 37 | 2 | 0 | 11 |
| | $III_2$ | 41·6 | 4·8 | 42·4 | 0 | 0 | 11·2 | 51 | 7 | 40 | 2 | 0 | 0 |
| 10 | I | 14·4 | 1·6 | 78·4 | 0·8 | 0 | 4·8 | 17 | 0 | 64 | 10 | 0 | 9 |
| | II | 28·8 | 0·8 | 46·4 | 17·6 | 0 | 6·4 | 16 | 0 | 62 | 13 | 5 | 4 |
| | III | 30·4 | 1·6 | 40·0 | 28·0 | 0 | 0 | 24 | 1 | 57 | 9 | 3 | 6 |
| 11 | $I_1$ | 7·2 | 6·4 | 80·8 | 0 | 0 | 5·6 | 17 | 0 | 76 | 0 | 0 | 7 |
| | $I_2$ | 23·2 | 8·8 | 53·6 | 0 | 0 | 14·4 | 18 | 0·5 | 67·5 | 4 | 0 | 40 |
| | $II_1$ | 12·8 | 8·0 | 44·0 | 0 | 0 | 35·2 | 21 | 0 | 62 | 10 | 0 | 7 |
| | $II_2$ | 24·0 | 8·0 | 56·0 | 0 | 0 | 12·0 | 20 | 2 | 67 | 0 | 0 | 12·0 |
| | $II_3$ | 32·0 | 1·6 | 53·6 | 0 | 0 | 12·8 | 11 | 0 | 82 | 0 | 0 | 7 |
| 12 | $I_1$ | 22·4 | 0 | 60·0 | 0 | 0 | 17·6 | 20·9 | 2·7 | 63·6 | 0 | 1·8 | 10·9 |
| | $I_2$ | 28·8 | 9·6 | 52·0 | 0 | 0 | 9·6 | 16·4 | 10·5 | 64·9 | 0 | 0·8 | 7·5 |
| | $I_3$ | 27·2 | 16·8 | 49·6 | 0 | 0 | 6·4 | 33·3 | 0 | 55·8 | 0 | 0 | 10·8 |
| 13 | I | | | | | | | 23 | 5 | 54 | 0 | 0 | 18 |
| | II | | | | | | | 32 | 2 | 52 | 0 | 0 | 14 |
| | $III_1$ | 4·0 | 9·6 | 72·8 | 0 | 0 | 13·6 | 8 | 8 | 71 | 0 | 0 | 13 |
| | $III_2$ | 14·4 | 8·8 | 55·2 | 0 | 0 | 21·6 | 6 | 6 | 76 | 0 | 0 | 12 |
| | IV | 30·4 | 4·0 | 60·0 | 0 | 0 | 5·6 | 30 | 2 | 59 | 0 | 0 | 9 |
| | $V_1$ | 29·6 | 6·4 | 60·0 | 0 | 0 | 4·0 | 16 | 7 | 59 | 0 | 0 | 18 |
| | $V_2$ | 32·8 | 6·4 | 52·8 | 0 | 0 | 8·0 | 14 | 4 | 68 | 0 | 0 | 14 |

* Primary tumour.

† Metastasis.

‡ Roman numbers denote individual pieces used for chemical analysis. Arabic numbers denote subsamples examined microscopically.

¶ T = tumour cells. N = necrotic tissue. F = fibrous tissue. A = adipose tissue. G = glandular tissue. O = other elements including empty space.

by the two methods for all the specimens received. The two different methods of scoring gave results for the percentage of tumour which are broadly similar. However, scores for different samples from the same subject by either counting method are characterized by considerable variability.

For the proportions of tumour cells, the mean difference between the two methods was 0·77 % and was not significantly different from zero. The between-tumour variance of the differences between the two methods was 1·72 times that of the within-tumour variance, but the ratio did not differ significantly from 1.

It is reasonable to conclude that although the histological estimation of the percentage of tumour may bear a relationship to the amount present in the whole tumour, there was considerable variation between different samples from the same subject.

I sincerely thank Professor Richard D. Remington, London School of Hygiene and Tropical Medicine and University of Michigan, for his kind advice and help with the statistical analysis of these results.